# Editorial: Novel mechanisms involved in urinary bladder control: Advances in neural, humoral and local factors underlying function and disease, volume II

**DOI:** 10.3389/fphys.2022.1056316

**Published:** 2022-10-17

**Authors:** Monica A. Sato, Laurival A. De Luca, Russ Chess-Williams, Patrik Aronsson

**Affiliations:** ^1^ Department of Morphology and Physiology, Faculdade de Medicina do ABC, Centro Universitario FMABC, Santo Andre, Brazil; ^2^ Department of Physiology and Pathology, Faculty of Dentistry, São Paulo State University, Araraquara, Brazil; ^3^ Faculty of Health Sciences & Medicine, Bond University, Gold Coast, QLD, Australia; ^4^ Department of Pharmacology, Institute of Neuroscience and Physiology, University of Gothenburg, Sahlgrenska Academy, Gothenburg, Sweden

**Keywords:** urinary bladder, overactive bladder, diabetes mellitus, purinergic, angiotensin-(1-7) [Ang-(1-7)], urofacial syndrome, HPSE2, brain network

This second volume of a Research Topic devoted to the investigation of the control of urinary bladder in physiological and pathological conditions reiterates its relevance. As a subject with several “facets” (Sato et al., 2020), not surprisingly, it brings new contributions of several researchers, thereby advancing further our knowledge about such control. The attentive reader will also see that the many facets continue to embrace a complete range of questions ranging from local organ mechanisms to those dependent on high brain functions. We are glad that both volumes, in addition to complete each other, also match in this respect.

The urine storage and voiding from the bladder are mediated by both central and peripheral mechanisms, nevertheless they still remain to be fully elucidated. Interestingly, Lamy et al. have shown that Angiotensin-(1–7) administrated intravenously or topically (*in situ*) onto the urinary bladder (UB) elicits an increase in the intravesical pressure. The authors also demonstrated that Mas receptors for Angiotensin-(1–7) and ACE-2, an enzyme required for Angiotensin-(1–7) synthesis, are expressed in the bladder. Therefore, they suggested that this peptide acts in the UB to increase the IP and can be also locally synthesized in the UB.

The review of Pang et al. presented in this topic shows previous functional imaging studies and combines them with brain regions involved in bladder control, demonstrating interactions between these regions, and brain networks, as well as changes in brain function in diseases affecting the urinary bladder. Pang et al. extend the working model proposed by Griffiths et al. (2015) about the brain network, and provide insights for current and future bladder-control research.

Several bladder diseases arise due to abnormal contractions (Chapple et al., 2018), and Phelps et al. aimed to identify the possible similarities in extracellular Ca2+ requirements between muscarinic, histamine, 5-hydroxytryptamine (5-HT), neurokinin-A (NKA), prostaglandin E2 (PGE2), and angiotensin II (ATII) receptors for mediating contractile activity of the urinary bladder (urothelium and lamina propria). Despite the finding that the specific requirement of Ca2+ on contractile responses varies depending on the receptor, Phelps et al. suggested that extracellular Ca2+ has a key role in mediating G protein-coupled receptor contractions of the urothelium and lamina propria.

Two of the studies examined purinergic mechanisms that operate within the bladder. The first investigated the role of purine metabolism in purinergic mechanisms. Earlier studies have shown that adenosine 5′-triphosphate (ATP) released from the urothelium has a prominent role in bladder mechanotransduction (Birder and Andersson, 2013; Takezawa et al., 2016). Urothelial ATP regulates the micturition cycle by activation of purinergic receptors, which are expressed in many cell types in the lamina propria (LP), including afferent neurons, and have been implicated in the direct mechanosensitive signaling between urothelium and detrusor (Cockayne et al., 2000; Vlaskovska et al., 2001; Burnstock, 2014). Aresta Branco et al. investigated possible mechanosensitive mechanisms of ATP hydrolysis in the LP at the anti-luminal side of nondistended (empty) or distended (full) murine (C57BL/6J) detrusor-free bladder model. The authors demonstrated that mechanosensitive degradation of ATP and ADP by membrane-bound and soluble nucleotidases in the LP reduces the availability of excitatory purines in the LP at the end of bladder filling. Hence, they suggested a possible safeguard mechanism to prevent overexcitability of the bladder, in which adequate proportions of excitatory and inhibitory purines in the bladder wall are determined by distention-associated purine release and purine metabolism.

The second purinergic study examined the role of P2X7 receptors in the bladder. The purinergic P2X7 receptor (P2X7R) is expressed abundantly on the bladder urothelium and its role in inflammation and cell death has been increasingly recognized (Vial and Evans, 2000; Menzies et al., 2003; Svennersten et al., 2015). It is well known that chemotherapy with cyclophosphamide can induce cystitis in the patients due to excretion of a toxic metabolite called acrolein. Cystitis is an inflammation of the bladder that is associated with damage to the integrity of the urothelial barrier. Taidi et al. investigated the role of P2X7R in acrolein-induced inflammatory damage in primary cultured porcine bladder urothelial cells. The authors demonstrated that acrolein induced a significant reduction in urothelial cell viability and barrier function, which was protected by the presence of P2X7R antagonist. Thereby, Taidi et al. suggested that P2X7R blockade may be a possible therapy in patients with bladder cystitis evoked by cyclophosphamide treatment.

The dysregulation in neurotransmission has been implicated in several lower urinary bladder conditions, however the mechanisms underlying the neurotransmitter release in the bladder still require elucidation. Carew et al. investigated the expression of myosin 5a (Myo5a), which is a motor protein that facilitates the directed motion of synaptic vesicles along actin fibers, in the regulation of excitatory neurotransmission in the bladder. The authors demonstrated that Myo5a is localized in cholinergic nerve fibers in the bladder and identified several Myo5a splice variants in the detrusor and suggested that the abundance of each is likely critical for efficient synaptic vesicle transport and neurotransmission in the bladder.

High glucose levels can induce changes in the urinary bladder. Oliveira et al. has shown that the treatment of mice with methylglyoxal (MGO), which is a compound generated during glycolysis and present in high levels in the plasma of patients with diabetes mellitus (Kilhovd et al., 2003; Han et al., 2009), induces detrusor overactivity through the formation of advanced glycation end products (AGE) that bind to RAGE receptors. These receptors are members of the immunoglobulin superfamily of cell surface receptors, responsible for recognizing endogenous ligands (Kim et al., 2021). Oliveira et al. also demonstrated that MGO treatment increased reactive oxygen species (ROS) production, which was markedly higher in the detrusor muscle than in the urothelium. They suggested that MGO accumulation increases AGE formation, which activates the RAGE-ROS signaling and consequent Rho kinase-induced muscle sensitization, which leads to detrusor overactivity.

Despite the urinary bladder is markedly enlarged in the streptozotocin-induced type 1 diabetes mellitus in rats, which may contribute to the frequent diabetic uropathy, very little is known about the bladder changes in type 2 diabetes models. Diabetic polyuria has been proposed as the pathophysiological mechanism behind bladder enlargement. In the review of Yesilyurt et al., bladder weight and blood glucose from 16 studies were evaluated and concluded that the presence and extent of bladder enlargement varied markedly depending on the diabetes models. The authors also suggest that particularly in type 2 diabetes models, the bladder enlargement is primarily driven by glucose levels/glucosuria.

Overactive bladder (OAB) has been accepted as an idiopathic disorder defined by urinary urgency, increased daytime urinary frequency and/or nocturia, with or without urinary incontinence. This clinical syndrome is characterized clinically by an absence of other organic diseases, including urinary tract infection. However, a growing body of evidence has shown that a significant proportion of OAB patients have active bladder infection. The review of Mansfield et al. discusses the findings of recent laboratory and clinical studies, providing the relationship between urinary tract infection, bladder inflammation, and the pathophysiology of OAB. The authors suggest that urinary tract infection may be an underappreciated contributor to the pathophysiology of some OAB patients who are resistant to standard treatments.

Urinary bladder function can also be affected by chronic psychological stress leading to an exacerbated lower urinary tract dysfunction as in OAB or interstitial cystitis-bladder pain syndrome (Macaulay et al., 1987; Lutgendorf et al., 2001; Rothrock et al., 2001; McVary et al., 2005; Fan et al., 2008; Zhang et al., 2013; Bradley et al., 2014; Lai et al., 2015a, 2015b). The review of Gao and Rodriguez highlights recent findings about stress-related animal models as water avoidance stress, social stress, early life stress, repeated variable stress, chronic variable stress, intermittent restraint stress and others, demonstrating that different types of chronic stress induce relatively distinguished changes at multiple levels of the micturition pathway.

This topic also brings a novelty showed by Beaman et al. about a rare disease called urofacial syndrome (UFS), which is an autosomal recessive congenital disorder of the urinary bladder characterized by voiding dysfunction and a grimace upon smiling (Elejalde, 1979; Ochoa 2004; Newman and Woolf, 2018; Osorio et al., 2021). Biallelic variants of the HPSE2 gene that encodes the secreted protein heparanase-2 have been described in about half of the families studied with UFS (McKenzie et al., 2000; Newman and Woolf, 2018; McKenzie 2020). Bladder autonomic neurons emerge from pelvic ganglia, in which resident neural cell bodies derive from migrating neural crest cells. Beaman et al. demonstrated in normal embryos that heparanase-2 and immunoglobulin like domains 2 (LRIG2) are expressed in neural like cells with a migratory phenotype, postulated to be pelvic ganglia precursors. Thereby, Beaman et al. suggested that biallelic variants of LRIG2 should be also implicated in the rare UFS.

In conclusion, this Research Topic encompasses a broad range of studies from basic science to clinical and certainly challenges researchers to further investigate unsolved questions. We trust that the valuable lessons learnt about the urinary bladder will be useful further for the development of novel therapeutic approaches upon the growing number of patients with bladder dysfunctions worldwide.
